# Salpingectomy and the Risk of Ovarian Cancer in Ontario

**DOI:** 10.1001/jamanetworkopen.2023.27198

**Published:** 2023-08-11

**Authors:** Vasily Giannakeas, Ally Murji, Lorraine L. Lipscombe, Steven A. Narod, Joanne Kotsopoulos

**Affiliations:** 1 Women’s College Research Institute, Women’s College Hospital, Toronto, Ontario, Canada; 2 Dalla Lana School of Public Health, University of Toronto, Toronto, Ontario, Canada; 3 ICES, Toronto, Ontario, Canada; 4 Department of Obstetrics and Gynecology, Mount Sinai Hospital, University of Toronto, Toronto, Ontario, Canada; 5 Department of Medicine, Faculty of Medicine, University of Toronto, Toronto, Ontario, Canada

## Abstract

**Importance:**

A body of pathological and clinical evidence supports the position that the fallopian tube is the site of origin for a large proportion of high-grade serous ovarian cancers. Consequently, salpingectomy is now considered for permanent contraception (in lieu of tubal ligation) or ovarian cancer prevention (performed opportunistically at the time of surgical procedures for benign gynecologic conditions).

**Objective:**

To evaluate the association between salpingectomy and the risk of invasive epithelial ovarian, fallopian tube, and peritoneal cancer.

**Design, Setting, and Participants:**

This population-based retrospective cohort study included all women aged 18 to 80 years who were eligible for health care services in Ontario, Canada. Participants were identified using administrative health databases from Ontario between January 1, 1992, and December 31, 2019. A total of 131 516 women were included in the primary (matched) analysis. Women were followed up until December 31, 2021.

**Exposures:**

Salpingectomy (with and without hysterectomy) vs no pelvic procedure (control condition) among women in the general population.

**Main Outcomes and Measures:**

Women with a unilateral or bilateral salpingectomy in Ontario between April 1, 1992, and December 31, 2019, were matched 1:3 to women with no pelvic procedure from the general population. Cox proportional hazards regression models were used to estimate the hazard ratios (HRs) and 95% CIs for ovarian, fallopian tube, and peritoneal cancer combined.

**Results:**

Among 131 516 women (mean [SD] age, 42.2 [7.6] years), 32 879 underwent a unilateral or bilateral salpingectomy, and 98 637 did not undergo a pelvic procedure. After a mean (range) follow-up of 7.4 (0-29.2) years in the salpingectomy group and 7.5 (0-29.2) years in the nonsurgical control group, there were 31 incident cancers (0.09%) and 117 incident cancers (0.12%), respectively (HR, 0.82; 95% CI, 0.55-1.21). The HR for cancer incidence was 0.87 (95% CI, 0.53-1.44) when comparing those with salpingectomy vs those with hysterectomy alone.

**Conclusions and Relevance:**

In this cohort study, no association was found between salpingectomy and the risk of ovarian cancer; however, this observation was based on few incident cases and a relatively short follow-up time. Studies with additional years of follow-up are necessary to define the true level of potential risk reduction with salpingectomy, although longer follow-up will also be a challenge unless collaborative efforts that pool data are undertaken.

## Key Points

**Question:**

Is salpingectomy associated with a lower risk of developing ovarian cancer?

**Findings:**

In this cohort study of 131 516 women in Ontario, there were 31 incident ovarian cancers among 32 879 women (0.09%) who had a salpingectomy compared with 117 incident ovarian cancers among 98 637 women (0.12%) who did not have a pelvic procedure.

**Meaning:**

These findings suggest no association between salpingectomy and the risk of ovarian cancer among women in the general population; given the rarity of this disease, additional follow-up is needed to reevaluate the potential association in an aging cohort.

## Teaser

This cohort study assesses the association between salpingectomy and the risk of invasive epithelial ovarian, fallopian tube, and peritoneal cancer among women in Ontario.

## Introduction

Epithelial ovarian cancer is the fifth leading cause of cancer death among women in Canada, with a 5-year survival rate of 45%.^1^ High-grade serous cancer is the most common subtype, typically presenting at an advanced stage; thus, the case fatality rate is high.^2^ There has been little progress in screening for early detection; apart from oral contraceptives, few factors have been reported to reduce risk or increase survival.^3^ Primary prevention with surgical procedures is only indicated for women at high risk of developing ovarian cancer.^4^ Although this disease is relatively rare, interventions that may lower the risk of developing ovarian cancer are necessary to reduce incidence and death.

Given the compelling molecular and pathological evidence supporting the fallopian tube as the site of origin for high-grade serous cancers, there has been a shift in the gynecologic community to replace tubal ligation with salpingectomy (removal of both fallopian tubes) for permanent contraception. Multiple organizations have recommended opportunistic bilateral salpingectomy at the time of surgical procedures for benign conditions (most commonly hysterectomy) for primary prevention of ovarian cancer.^5,6,7,8,9^ A few observational studies included in a meta-analysis^10^ have reported on the association between salpingectomy and ovarian cancer and have collectively suggested a 49% to 65% reduction in risk. Although limited, previous studies^11,12^ have reported no association of salpingectomy with ovarian function or morbidity. Findings regarding whether salpingectomy impacts mortality will not be available for several years given that salpingectomy in lieu of tubal ligation (or otherwise) was introduced into clinical practice guidelines around 2015.^13^

We conducted a population-based cohort study using health care administrative databases to report patterns of salpingectomy and evaluate the association between salpingectomy and the risk of invasive epithelial ovarian, fallopian tube, and peritoneal cancer (hereinafter, ovarian cancer). We compared cancer incidence among women who underwent salpingectomy (with and without hysterectomy) and women who did not undergo salpingectomy, and we compared the clinical characteristics of the cases diagnosed among women with and without a salpingectomy.

## Methods

### Study Design and Data Sources

This retrospective population-based matched cohort study used health care administrative databases in Ontario, Canada, which has a population of 14.5 million residents eligible for health care services under the province’s universal single-payer health care coverage. Data sets were linked using distinct encoded identifiers and analyzed at ICES, an independent nonprofit research institute whose legal status under Ontario’s health information privacy law allows it to collect and analyze health care and demographic data, without consent, for health system evaluation and improvement. The data sources and variable codes included in this analysis are shown in eTable 1 in Supplement 1. This study was approved by the research ethics board of Women’s College Hospital. Given the study’s retrospective nature, the board waived the requirement for informed consent. This study was performed in accordance with the Declaration of Helsinki.^14^ The study followed the Strengthening the Reporting of Observational Studies in Epidemiology (STROBE)^15^ and the Reporting of Studies Conducted Using Observational Routinely Collected Health Data (RECORD)^16^ reporting guidelines for cohort studies.

### Construction of Cohorts

#### Surgical Cohort

The inclusion cohort consisted of all women aged 18 to 80 years who were eligible for health care services in Ontario. Participants were identified using administrative health databases from Ontario between January 1, 1992, and December 31, 2019. Participants were followed up until December 31, 2021. A total of 131 516 women were included in the primary (matched) analysis.

Women were observed from the date of cohort entry to the first salpingectomy and/or hysterectomy. The date of the first operation was considered the reference date. The index date was defined as 180 days after the first operation to account for lead time that may have existed because of the operation and to avoid inclusion of occult cases as events. Participants were assigned to 1 of 3 mutually exclusive groups (salpingectomy only, salpingectomy with hysterectomy, or hysterectomy only) based on surgical procedures that occurred in the 180-day period after the first operation. Participants were excluded if, on the index date, they were ineligible for the Ontario Health Insurance Plan at any point in the previous 2.5 years or had a history of any of the following before the index date: any cancer diagnosis, precancerous ovarian condition, ovarian cysts, radical gynecologic operation, or previous oophorectomy (eTable 2 in Supplement 1).

We similarly created a tubal ligation cohort; women in this cohort were observed from the date of cohort entry to the first tubal ligation that occurred within the accrual period. The index date was defined as 180 days after tubal ligation. Women were excluded based on the same criteria listed in the previous paragraph or if they had a history of hysterectomy or salpingectomy.

#### Nonsurgical Control Cohort

To identify a cohort of women that could serve as a nonsurgical control group, we randomly assigned index dates to all participants in the inclusion cohort. Index dates were assigned based on the distribution of index dates among all eligible women in the salpingectomy group. Women were excluded if they had a history of salpingectomy or hysterectomy before their index date. Women in the surgical cohorts were eligible to serve in the nonsurgical control cohort if their randomly assigned index date preceded their surgical date.

### Covariates

We collected information on a series of variables that describe demographic information, health services use, reproductive history, comorbidities, and indications for surgery. Demographic variables included neighborhood income quintile, residence location, and years eligible for provincial health coverage. Health services use included history of core primary care visits, specialist visits, inpatient hospitalizations, and emergency department visits. Patient comorbidities were assessed using the ACG System, version 10.0 (The Johns Hopkins University), to capture aggregate diagnosis groups based on health services use in the 2 years before a participant’s reference date.^6^

### Matching

We created 3 models using propensity score methods to compare the various groups. Model 1 compared the salpingectomy (with and without hysterectomy) group with the nonsurgical control cohort. Patients who underwent a unilateral or bilateral salpingectomy in Ontario between April 1, 1992, and December 31, 2019, were matched 1:3 to women who did not undergo a gynecologic procedure. Participants were matched on year of index date, age at index date (plus or minus 2 years), parity, history of tubal ligation, and propensity score.

Model 2 compared the salpingectomy (with and without hysterectomy) group with the hysterectomy only group. This model evaluated the difference in association between salpingectomy and a surgical comparator. All patients who underwent a salpingectomy were matched 1:1 to patients who underwent a hysterectomy alone.

Model 3 compared the tubal ligation group with the nonsurgical control cohort. Participants who underwent tubal ligation were matched 1:3 to women who did not undergo a gynecologic procedure. Participants who underwent tubal ligation were matched using the same methods and variables as those used in model 1; however, participants in the matched nonsurgical control cohort could not have a history of tubal ligation.

### Outcomes

#### Primary Outcomes

The primary outcome was incident invasive ovarian cancer (including epithelial ovarian, fallopian tube, or peritoneal cancer) documented in the Ontario Cancer Registry during the follow-up period (diagnosis and procedure codes are shown in eTable 1 in Supplement 1). Matched participants were followed up from their index date to the first of a primary outcome event, death, end of eligibility for the Ontario Health Insurance Plan, oophorectomy, or December 31, 2021.

#### Tracer Outcomes

Tracer events are outcomes expected to have no association with the exposure variable. An association with these outcomes may suggest the presence of residual confounding or bias. We selected 2 cancer-related tracer outcomes, incident breast cancer and incident lung cancer, that we suspected would not be associated with salpingectomy. We used the Ontario Cancer Registry to capture these incident cancers as tracer events. Participants were followed up using the same approach as that used for the primary outcomes.

#### Sensitivity Analysis

In a sensitivity analysis, we censored women who underwent a gynecologic procedure of interest in the follow-up period that may have biased the effect estimates. Specifically, no participants in the nonsurgical control cohort and the tubal ligation group were censored if they underwent a salpingectomy or hysterectomy in the follow-up period, while women who had undergone a salpingectomy without a hysterectomy were censored if they had a hysterectomy in the follow-up period. We also limited the cohort to patients who had only bilateral salpingectomy by excluding those with a unilateral salpingectomy and those for whom laterality was unknown.

### Statistical Analysis

Baseline descriptive characteristics of the surgical and nonsurgical cohorts were compared using standardized differences. Analysis of variance and Kruskal-Wallis tests were used to compare continuous variables, and the χ^2^ test was used to compare categorical variables, across 2 or more groups. A standardized difference of less than 0.10 was used to determine comparability between the groups for each covariate of interest.^17^ Kaplan-Meier analysis was used to estimate the cumulative incidence of cancer among matched participants. Crude incident rates of cancer were calculated for each group by dividing the number of outcome events by the total number of person-years in the follow-up period. Cox proportional hazards regression models were used to estimate the adjusted hazard ratios (HRs) and 95% CIs for each exposure group. The threshold for statistical significance was 2-tailed *P* = .05. All statistical analyses were performed using SAS software, version 9.4 (SAS Institute Inc). A more detailed description of the methods is available in the eMethods in Supplement 1. We performed a post hoc sample size calculation to estimate the number of ovarian cancer events needed to detect an HR of 0.80 for our primary analysis at 80% power and α = .05.

## Results

Among 131 516 women included in the analyses, the mean (SD) age was 42.2 (7.6) years. Of those, 32 879 received a unilateral or bilateral salpingectomy (with and without hysterectomy), and 98 637 did not receive a surgical procedure.

**Figure 1.**
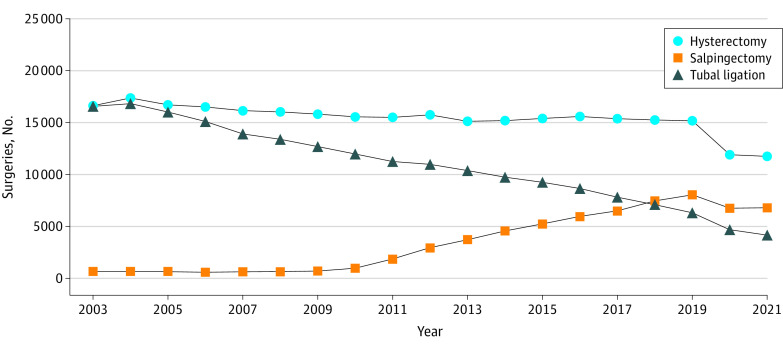
Frequencies of Hysterectomy, Salpingectomy, and Tubal Ligation in Ontario From 2003 to 2021 Information on procedure type was obtained from the Canadian Institute for Health Information Discharge Abstract database for inpatient procedures and the Same-Day Surgery database for outpatient procedures.

To evaluate patterns in procedures from 2003 to 2021, we combined all women with a salpingectomy (irrespective of hysterectomy status and location of procedure). We found a decrease in tubal ligation starting in 2004 (from 16 811 procedures in 2004 to 6311 in 2019) and an increase in salpingectomy predominantly after 2010 (from 975 procedures in 2010 to 8060 in 2019) (Figure 1).

A total of 13 451 women in the salpingectomy only group were compared with 20 842 women in the salpingectomy with hysterectomy group. Significant differences in baseline patient characteristics were observed between the 2 exposed groups (Table 1). For example, patients in the salpingectomy only vs salpingectomy with hysterectomy group were younger (mean [SD] age, 38.7 [7.9] years vs 44.2 [6.6] years; *P* < .001) and underwent fewer bilateral procedures (8739 [65.0%] vs 17 717 [85.0%]; *P* < .001). There were 16 cases (0.12%) of ovarian cancer in the salpingectomy only group vs 15 cases (0.07 %) in the salpingectomy with hysterectomy group (*P* = .16). After matching, participants in the surgical groups were similar to those in the nonsurgical control cohort with respect to demographic characteristics, health services use, and comorbidities (eg, eTable 3 in Supplement 1).

**Table 1.** Characteristics of Women Undergoing Salpingectomy, Overall and by Hysterectomy Status

Characteristic	Patients, No. (%)			*P* value for salpingectomy with hysterectomy vs salpingectomy without hysterectomy^a^
	Salpingectomy with and without hysterectomy (n = 34 293)	Salpingectomy with hysterectomy (n = 20 842)	Salpingectomy without hysterectomy (n = 13 451)	
Calendar year				
Mean (SD)^b^	2013.4 (7.2)	2014.3 (6.1)	2011.9 (8.3)	<.001
Median (IQR) [range]	2016 (2012-2018) [1992-2020]	2016 (2013-2018) [1992-2020]	2016 (2007-2018) [1992-2020]	<.001
Age, y				
Mean (SD)	42.0 (7.7)	44.2 (6.6)	38.7 (7.9)	<.001
Median (IQR) [range]	42.3 (37.0-46.9) [18.0-79.8]	44.5 (40.2-48.1) [18.3-79.8]	38.1 (33.4-43.1) [18.0-79.8]	<.001
Group				
18-29	1943 (5.7)	362 (1.7)	1581 (11.8)	<.001
30-39	11 194 (32.6)	4629 (22.2)	6565 (48.8)	
40-49	17 312 (50.5)	12 918 (62.0)	4394 (32.7)	
50-59	3271 (9.5)	2577 (12.4)	694 (5.2)	
60-69	385 (1.1)	241 (1.2)	144 (1.1)	
70-80	188 (0.5)	115 (0.6)	73 (0.5)	
Neighborhood income quintile				
1 (lowest)	6601 (19.2)	3673 (17.6)	2928 (21.8)	<.001
2	6880 (20.1)	4101 (19.7)	2779 (20.7)	
3	7096 (20.7)	4410 (21.2)	2686 (20.0)	
4	7226 (21.1)	4512 (21.6)	2714 (20.2)	
5 (highest)	6377 (18.6)	4081 (19.6)	2296 (17.1)	
Missing	113 (0.3)	65 (0.3)	48 (0.4)	
Residence location				
Urban	29 652 (86.5)	18 047 (86.6)	11 605 (86.3)	.55
Rural	4587 (13.4)	2765 (13.3)	1822 (13.5)	
Missing	54 (0.2)	30 (0.1)	24 (0.2)	
Core primary care visits with FP or GP				
Mean (SD)	3.8 (4.1)	3.7 (4.1)	3.9 (4.0)	.01
Median (IQR) [range]	3.0 (1.0-5.0) [0-112.0]	3.0 (1.0-5.0) [0-88.0]	3.0 (1.0-5.0) [0-112.0]	<.001
No. of urgent inpatient hospitalization episodes				
0	31 618 (92.2)	19 599 (94.0)	12 019 (89.4)	<.001
1	2135 (6.2)	1014 (4.9)	1121 (8.3)	
2	375 (1.1)	158 (0.8)	217 (1.6)	
≥3	165 (0.5)	71 (0.3)	94 (0.7)	
No. of ADGs				
Mean (SD)	7.0 (2.9)	7.0 (2.9)	7.0 (3.0)	.57
Median (IQR) [range]	7 (5-9) [0-21]	7 (5-9) [0-20]	7 (5-9) [0-21]	.45
Group				
0-4	7235 (21.1)	4319 (20.7)	2916 (21.7)	.008
5-9	20 534 (59.9)	12 617 (60.5)	7917 (58.9)	
≥10	6524 (19.0)	3906 (18.7)	2618 (19.5)	
Laterality of surgical procedure				
Bilateral	26 456 (77.1)	17 717 (85.0)	8739 (65.0)	<.001
Unilateral	3468 (10.1)	1414 (6.8)	2054 (15.3)	
Unknown	4369 (12.7)	1711 (8.2)	2658 (19.8)	
Indications at surgical procedure				
Abnormal bleeding	15 329 (44.7)	13 458 (64.6)	1871 (13.9)	<.001
Fibroids	12 764 (37.2)	10 981 (52.7)	1783 (13.3)	<.001
Endometriosis	6275 (18.3)	5016 (24.1)	1259 (9.4)	<.001
Prolapse	2154 (6.3)	1956 (9.4)	198 (1.5)	<.001
Pelvic pain or inflammation	9596 (28.0)	5799 (27.8)	3797 (28.2)	.41
Ectopic pregnancy	1153 (3.4)	13 (0.1)	1140 (8.5)	<.001
Sterilization	6220 (18.1)	57 (0.3)	6163 (45.8)	<.001
Prophylactic procedure	647 (1.9)	92 (0.4)	555 (4.1)	<.001
Oophorectomy in follow-up period	1419 (4.1)	616 (3.0)	803 (6.0)	<.001
Follow-up time, y				
Mean (SD)	7.4 (6.5)	6.7 (5.6)	8.4 (7.6)	<.001
Median (IQR) [range]	5.2 (3.1-8.4) [0-29.2]	5.2 (3.2-7.9) [0-29.2]	5.4 (3.0-10.4) [0-29.2]	<.001
Incident cancers				
Ovarian^c^	31 (0.09)	15 (0.07)	16 (0.12)	.16
Breast	446 (1.30)	268 (1.29)	178 (1.32)	.77
Lung	88 (0.26)	53 (0.25)	35 (0.26)	.92

Abbreviations: ADG, aggregate diagnosis group; FP, family practitioner; GP, general practitioner.

^a^ Analysis of variance and Kruskal-Wallis test were used for continuous variables, and χ^2^ test was used for categorical variables.

^b^ Decimal values represent the proportion of the year (eg, 2013.5 is approximately 182 days into 2013).

^c^ Includes invasive epithelial ovarian, fallopian tube, and primary peritoneal cancer.

**Figure 2.**
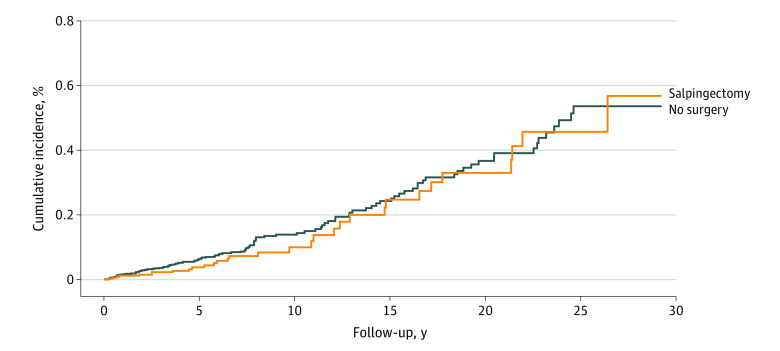
Cumulative Incidence of Ovarian Cancer in Ontario Among Women Undergoing Salpingectomy vs No Surgical Procedure Ovarian cancer includes invasive epithelial ovarian, fallopian tube, and primary peritoneal cancer. Log-rank *P* = .31.

The risk of composite ovarian cancer according to analytic model is shown in Table 2. There were 31 incident cancers (0.09%) in the salpingectomy (with and without hysterectomy) group and 117 (0.12%) in the nonsurgical control cohort. A nonsignificant 18% reduction in risk was observed for the salpingectomy (with and without hysterectomy) group (n = 32 879) compared with the nonsurgical control cohort (n = 98 637; HR, 0.82; 95% CI, 0.55-1.21; *P* = .31; mean [range] follow-up, 7.4 [0-29.2] years vs 7.5 [0-29.2] years). A nonsignificant 13% reduction in risk was observed for the salpingectomy (with and without hysterectomy) group (n = 21 724) vs the hysterectomy-only group (n = 21 724; HR, 0.87; 95% CI, 0.53-1.44; *P* = .59; mean [range] follow-up, 9.0 [0-29.2] years vs 9.2 [0-29.2] years). There was a 23% significant decrease in the risk of cancer among women who had a tubal ligation (n = 141 698) compared with women who did not have a pelvic procedure (n = 425 094; HR, 0.77; 95% CI, 0.64-0.93; *P* = .006; mean [range] follow-up, 12.5 [0-29.2] years vs 12.6 [0-29.3] years). The cumulative incidence of cancer for women in the salpingectomy (with and without hysterectomy) group compared with the nonsurgical control cohort is shown in Figure 2. The cumulative incidence of cancer for women in the tubal ligation group compared with the nonsurgical control cohort is shown in the eFigure in Supplement 1.

**Table 2.** Risk of Ovarian Cancer Among Women With Salpingectomy or Tubal Ligation by Analytic Model^a^

Model^b^	Patients, No.	Person-years	Follow-up, mean (range), y	Events, No.	Rate per 100 000 person-years	HR (95% CI)	*P* value
Model 1^c^							
No surgical procedure	98 637	742 837	7.5 (0-29.2)	117	15.75	1 [Reference]	.31
Salpingectomy with and without hysterectomy	32 879	242 691	7.4 (0-29.2)	31	12.77	0.82 (0.55-1.21)	
Model 2^d^							
Hysterectomy alone	21 724	199 986	9.2 (0-29.2)	33	16.50	1 [Reference]	.59
Salpingectomy with and without hysterectomy	21 724	196 344	9.0 (0-29.2)	28	14.26	0.87 (0.53-1.44)	
Model 3^e^							
No surgical procedure	425 094	5 341 582	12.6 (0-29.3)	557	10.43	1 [Reference]	.006
Tubal ligation	141 698	1 767 143	12.5 (0-29.2)	142	8.04	0.77 (0.64-0.93)	

Abbreviation: HR, hazard ratio.

^a^ Ovarian cancer includes invasive epithelial ovarian, fallopian tube, and primary peritoneal cancer.

^b^ Participants were matched on year of index date, age at index date (plus or minus 2 years), parity, history of tubal ligation, and propensity score.

^c^ Model 1 compared the salpingectomy (with and without hysterectomy) group with the nonsurgical control group. All patients who received salpingectomy were matched 1:3 to women who did not receive a pelvic procedure. This propensity score model incorporated income quintile, rurality of residence, years eligible for provincial health coverage, number of primary care visits, history of supracervical hysterectomy, and individual aggregate diagnosis groups.

^d^ Model 2 compared the salpingectomy (with and without hysterectomy) group with the hysterectomy-only group. All patients who received salpingectomy were matched 1:1 to patients who received hysterectomy only. The propensity score also included variables for surgical indications. Participants were caliper matched on a value that was 0.2 times the SD of the logit of the propensity score.

^e^ Model 3 compared the tubal ligation group with the nonsurgical control group. Patients who received tubal ligation were matched 1:3 to women who did not receive a pelvic procedure.

In the sensitivity analysis limited to invasive epithelial ovarian cancer only (excluding fallopian tube and peritoneal cancer), findings did not change substantially among those in the salpingectomy (with and without hysterectomy) group vs the nonsurgical control cohort (HR, 0.81; 95% CI, 0.54-1.22; *P* = .32) (eTable 4 in Supplement 1). Results of the sensitivity analysis with additional censoring for a gynecologic procedure in the follow-up period also revealed no association with cancer risk (HR, 0.84; 95% CI, 0.56-1.24; *P* = .38) (eTable 5 in Supplement 1).

In a post hoc analysis, we excluded matched pairs with a unilateral salpingectomy or unknown laterality. Among women with a bilateral salpingectomy (n = 25 409), there were 11 cases (0.04%) of cancer diagnosed, with a mean follow-up of 5.2 years (range, 0-19.2 years). Among women in the nonsurgical control cohort (n = 76 227), there were 60 cases (0.08%) of cancer diagnosed, with a mean follow-up of 5.2 years (range, 0-20.0 years). The HR for bilateral salpingectomy compared with no surgical procedure was 0.55 (95% CI, 0.29-1.05; *P* = .07), representing a 45% decrease in risk; however, this reduction was not statistically significant (eTable 6 in Supplement 1).

Among cancer cases in the nonsurgical control cohort (n = 110), 45 (40.91%) were serous and 65 (59.09%) were nonserous or missing compared with 15 (51.72%) serous and 14 (48.28%) nonserous or missing in the salpingectomy (with and without hysterectomy) group (n = 29) (eTable 4 in Supplement 1). We could not report on stage of disease among the cases given the high rates of missingness for this variable.

## Discussion

In this large population-based cohort study with 7 years of follow-up, salpingectomy did not confer a statistically significant protective benefit for ovarian cancer compared with no surgical procedure. Our point estimate revealed an 18% nonsignificant reduction in risk in the salpingectomy (with and without hysterectomy) group (HR, 0.82) and a similar level of nonsignificant risk reduction with the inclusion of a hysterectomy comparator group (HR, 0.87). When limited to patients with a documented bilateral salpingectomy, the protective benefit was greater (HR, 0.55) despite a follow-up period of only 5.2 years, although the result was not significant. Tubal ligation, which is a bilateral procedure, was associated with a significant 23% reduction in risk (HR, 0.77); however, the follow-up period among women who received tubal ligation was substantially longer (mean, 12.5 years), and the number of women in the tubal ligation group was considerably larger (141 698 women vs 25 409 in the bilateral salpingectomy group).

Even with inclusion of a large population, the analysis remained underpowered to detect a statistically significant difference given the rarity of our end point of interest and the relatively short follow-up period in the salpingectomy group. In a post hoc sample size calculation, we estimated that 636 events (exposed and unexposed groups) were needed to detect an HR of 0.80 for our primary analysis (ie, model 1) at 80% power and α = .05. The time required for the data to mature to detect a difference is lengthy, and this longer follow-up period will also be a challenge unless collaborative efforts that combine data from large-scale observational studies are made.

The shift to salpingectomy is a recent clinical phenomenon (Figure 1). Clinical practice guidelines are now recommending salpingectomy instead of tubal ligation (or at the time of another surgical procedure) based on the potential to prevent a subset of cancers originating in the fallopian tubes rather than the ovaries. The substantial (albeit nonsignificant) 45% reduction in risk in the analysis restricted to women with a known bilateral procedure is notable. The safety and acceptability of this procedure has been established,^12,18,19,20,21^ and, although the implications for ovarian function are less defined, studies^11,22,23^ have found no association with ovarian reserve or indicators of menopausal onset. Furthermore, Naumann et al^24^ recently estimated that universal opportunistic salpingectomy may prevent deaths from ovarian cancer and reduce health care costs. Thus, a clear translational aspect of the experimental evidence is to offer bilateral salpingectomy to women at average population risk with the aim of preventing the most aggressive form of this rare but fatal disease.^25,26^

To our knowledge, only 4 other studies^27,28,29,30^ have also evaluated the association of salpingectomy with ovarian cancer risk. The first was a case-control study of 194 women with serous ovarian or primary peritoneal cancer and 388 women without either diagnosis.^27^ Lessard-Anderson et al^27^ reported a nonsignificant 64% decrease in risk among women who underwent excisional tubal sterilization compared with women who did not undergo sterilization and women who underwent nonexcisional tubal sterilization (odds ratio [OR], 0.36; 95% CI, 0.13-1.02). There was no association between any tubal sterilization procedure and risk of cancer (OR, 0.59; 95% CI, 0.29-1.17).^27^ The researchers included complete salpingectomy, distal fimbriectomy, and partial salpingectomy in the exposed group; we included all procedure codes associated with a potential salpingectomy.

Madsen et al^28^ used a Danish nationwide registry to evaluate the association of tubal ligation and salpingectomy with cancer risk (13 241 women in the epithelial ovarian cancer group and 194 689 in the control group). They found a significant 42% decrease in risk with bilateral salpingectomy vs no salpingectomy (OR, 0.58; 95% CI, 0.36-0.95) but no association with unilateral salpingectomy (OR, 0.90; 95% CI, 0.72-1.12)^28^; however, these estimates were based on 17 women who underwent bilateral salpingectomy and 89 women who underwent unilateral salpingectomy, and the control group included women who underwent hysterectomy and/or tubal ligation.

In a study from Sweden that used an analytic approach similar to ours, Falconer et al^29^ reported a significant 65% decrease in the risk of ovarian or fallopian tube cancer with bilateral salpingectomy for benign conditions compared with an unexposed population. There were 7 cases of ovarian cancer among the 3051 women (0.23%) who underwent bilateral salpingectomy vs 30 682 cases among the 5 449 119 women (0.56%) in the unexposed group (HR, 0.35; 95% CI, 0.17-0.73).^29^ Unilateral salpingectomy was also associated with a significant (albeit less substantial) reduction in risk (HR, 0.71; 95% CI, 0.56-0.91).^29^ Several reasons may explain the differences in association found in the study by Falconer et al^29^ vs our study, including (1) longer follow-up period (mean, 18.0 years in the salpingectomy group and 23.1 years in the unexposed group^29^ vs 7.4 years and 7.5 years in our study), (2) younger age at cohort entry (eg, salpingectomy group: mean, 35.7 years^29^ vs 42.0 years in our study), and (3) higher ovarian cancer incidence rates in both the exposed and unexposed groups (0.23% and 0.56%, respectively,^29^ vs 0.09% and 0.12% in our study).

Hanley et al^30^ recently reported expected vs observed rates of ovarian cancer among women who underwent opportunistic salpingectomy in British Columbia between 2008 and 2017. The exposed group only included women who had fallopian tubes removed for the purpose of sterilization or at the time of hysterectomy. Using age-adjusted rates of ovarian cancer from the control group, they reported no cases of serous ovarian cancer vs 5.27 expected cases (and ≤5 cases of total epithelial ovarian cancers vs 8.68 expected cases) in the salpingectomy group.^30^ This number of cases was substantially lower than expected. Hanley et al^30^ concluded that opportunistic salpingectomy was a beneficial primary prevention strategy at the population level; however, future studies with more follow-up time were needed to provide more robust and definitive conclusions.

### Strengths and Limitations

This study has several strengths. A key strength was the use of validated administrative databases in a large population, reflecting the landscape in a province with universal health care. The ability to link to provincial registries ensured complete data on both exposures and outcomes and avoided the impact of recall bias that can occur with the use of self-reported data. Our strict matching criteria ensured similarities across the comparison groups. Furthermore, our consistent findings in the sensitivity analyses with additional censoring suggests a robust statistical approach. Although we did not directly assess the impact of opportunistic salpingectomy, our exclusion and censoring criteria ensured that women undergoing surgical procedures for potential tumor, precancerous conditions, or ovarian cysts were excluded.

The study also has limitations. We did not have detailed information on various risk factors for ovarian cancer (eg, hormone use, family history of disease, or germline variant); however, there is no reason to expect differences in these potential confounders and type of procedure. Lack of indication for a surgical procedure may have resulted in selection bias, with women who had higher baseline risk more likely to have undergone a salpingectomy; however, this higher likelihood of undergoing salpingectomy would have attenuated any benefits for our outcome of interest. The analyses of cancer risk were not sufficiently powered, particularly to evaluate heterogeneity by site of origin or histological subtype given the rarity of this disease and short follow-up time.

## Conclusions

This cohort study found an increase in the rates of salpingectomy over time, with a corresponding decrease in the rates of tubal ligation, among women in Ontario. Although the primary analysis was not sufficiently powered, the level of risk reduction with salpingectomy was similar to that observed with tubal ligation. This finding suggests that if removal of healthy fallopian tubes truly reduces the risk of ovarian cancer, future studies (with additional years of follow-up) should reveal a significant and clinically meaningful decrease in cases. However, the current study found no significant decrease in ovarian cancer rates in Ontario despite the increase in salpingectomy between 2003 and 2021. Given the rarity of this disease, additional follow-up is needed to reevaluate the potential association in an aging cohort. The increasing uptake of salpingectomy may offer an opportunity to prevent a proportion of cancers putatively arising from the fallopian tube and impact the mortality rates associated with a disease with a poor outcome.

## Notes

Supplement 1. **eMethods.** Study Design, Population, and Data; Construction of Cohort; Covariates; Matching; Outcomes; and Statistical Analysis **eTable 1.** Data Sources and Detailed Variable Definitions **eTable 2.** Inclusions and Exclusions **eTable 3.** Descriptive Characteristics of Matched Participants Included in Analysis of Salpingectomy Without Hysterectomy vs No Surgical Procedure **eTable 4.** Hazard Ratios and 95% CIs of Ovarian Cancer by Analytic Model **eTable 5.** Hazard Ratios and 95% CIs of Ovarian Cancer by Analytic Model, With Additional Censoring **eTable 6.** Hazard Ratios and 95% CIs of Ovarian Cancer Among Women With a Bilateral Salpingectomy by Analytic Model **eFigure.** Cumulative Incidence of Ovarian Cancer in Patients With Tubal Ligation vs No Surgical Procedure

Supplement 2.
Data Sharing Statement

## References

[1] Canadian Cancer Society; Canadian cancer statistics 2022. November 2022. Accessed March 2023. https://cancer.ca/en/research/cancer-statistics/canadian-cancer-statistics.

[2] Sopik V, Iqbal J, Rosen B, Narod SA. Why have ovarian cancer mortality rates declined? part II. case-fatality. Gynecol Oncol. 2015;138(3):750-756. doi:10.1016/j.ygyno.2015.06.016 26080288

[3] Beral V, Doll R, Hermon C, Peto R, Reeves G; Collaborative Group on Epidemiological Studies of Ovarian Cancer. Ovarian cancer and oral contraceptives: collaborative reanalysis of data from 45 epidemiological studies including 23,257 women with ovarian cancer and 87,303 controls. Lancet. 2008;371(9609):303-314. doi:10.1016/S0140-6736(08)60167-1 18294997

[4] Daly MB, Pal T, Berry MP, . Genetic/familial high-risk assessment: breast, ovarian, and pancreatic, version 2.2021, NCCN Clinical Practice Guidelines in Oncology. J Natl Compr Canc Netw. 2021;19(1):77-102. doi:10.6004/jnccn.2021.0001 33406487

[5] American College of Obstetricians and Gynecologists. ACOG Committee opinion No. 774: opportunistic salpingectomy as a strategy for epithelial ovarian cancer prevention. Obstet Gynecol. 2019;133(4):e279-e284. doi:10.1097/AOG.0000000000003164 30913199

[6] American College of Obstetricians and Gynecologists. ACOG Committee opinion No. 774 summary: opportunistic salpingectomy as a strategy for epithelial ovarian cancer prevention. Obstet Gynecol. 2019;133(4):842-843. doi:10.1097/AOG.0000000000003165 30913193

[7] Runnebaum IB, Kather A, Sehouli J. Opportunistic salpingectomy for the primary prevention of ovarian cancer. Dtsch Arztebl Int. 2022;119(49):846-847. doi:10.3238/arztebl.m2022.0232 36814422 PMC9981983

[8] Salvador S, Scott S, Francis JA, Agrawal A, Giede C. No. 344—opportunistic salpingectomy and other methods of risk reduction for ovarian/fallopian tube/peritoneal cancer in the general population. J Obstet Gynaecol Can. 2017;39(6):480-493. doi:10.1016/j.jogc.2016.12.005 28527613

[9] Subramaniam A, Einerson BD, Blanchard CT, . The cost-effectiveness of opportunistic salpingectomy versus standard tubal ligation at the time of cesarean delivery for ovarian cancer risk reduction. Gynecol Oncol. 2019;152(1):127-132. doi:10.1016/j.ygyno.2018.11.009 30477808 PMC6321779

[10] Yoon SH, Kim SN, Shim SH, Kang SB, Lee SJ. Bilateral salpingectomy can reduce the risk of ovarian cancer in the general population: a meta-analysis. Eur J Cancer. 2016;55:38-46. doi:10.1016/j.ejca.2015.12.003 26773418

[11] Hanley GE, Kwon JS, McAlpine JN, Huntsman DG, Finlayson SJ, Miller D. Examining indicators of early menopause following opportunistic salpingectomy: a cohort study from British Columbia, Canada. *Am J Obstet Gynecol*. 2020;223(2):221.e1-221.e11. doi:10.1016/j.ajog.2020.02.005 32067967

[12] Kim AJ, Barberio A, Berens P, . The trend, feasibility, and safety of salpingectomy as a form of permanent sterilization. J Minim Invasive Gynecol. 2019;26(7):1363-1368. doi:10.1016/j.jmig.2019.02.003 30771489

[13] Society of Gynecologic Oncology. SGO clinical practice statement: salpingectomy for ovarian cancer prevention. November 1, 2013. Accessed July 20, 2023. https://www.sgo.org/clinical-practice/guidelines/sgo-clinical-practice-statement-salpingectomy-for-ovarian-cancer-prevention/

[14] World Medical Association. World Medical Association Declaration of Helsinki: ethical principles for medical research involving human subjects. *JAMA*. 2013;310(20):2191-2194. doi:10.1001/jama.2013.281053 24141714

[15] von Elm E, Altman DG, Egger M, Pocock SJ, Gøtzsche PC, Vandenbroucke JP; STROBE Initiative. The Strengthening the Reporting of Observational Studies in Epidemiology (STROBE) statement: guidelines for reporting observational studies. Ann Intern Med. 2007;147(8):573-577. doi:10.7326/0003-4819-147-8-200710160-00010 17938396

[16] Benchimol EI, Smeeth L, Guttmann A, ; RECORD Working Committee. The Reporting of Studies Conducted Using Observational Routinely-Collected Health Data (RECORD) statement. PLoS Med. 2015;12(10):e1001885. doi:10.1371/journal.pmed.1001885 26440803 PMC4595218

[17] Austin PC, Grootendorst P, Anderson GM. A comparison of the ability of different propensity score models to balance measured variables between treated and untreated subjects: a Monte Carlo study. Stat Med. 2007;26(4):734-753. doi:10.1002/sim.2580 16708349

[18] Hanley GE, Kwon JS, Finlayson SJ, Huntsman DG, Miller D, McAlpine JN. Extending the safety evidence for opportunistic salpingectomy in prevention of ovarian cancer: a cohort study from British Columbia, Canada. *Am J Obstet Gynecol*. 2018;219(2):172.e1-172.e8. doi:10.1016/j.ajog.2018.05.019 29852159

[19] Hanley GE, Rozenberg NMK, McAlpine JN. Risk-reducing surgery in women at low lifetime risk of developing ovarian carcinoma: opportunistic salpingectomy. Clin Obstet Gynecol. 2017;60(4):758-770. doi:10.1097/GRF.0000000000000315 28957952

[20] Hanley GE, McAlpine JN, Pearce CL, Miller D. The performance and safety of bilateral salpingectomy for ovarian cancer prevention in the United States. *Am J Obstet Gynecol*. 2017;216(3): 270.e1-270.e9. doi:10.1016/j.ajog.2016.10.035 27810554

[21] Dilley SE, Havrilesky LJ, Bakkum-Gamez J, . Cost-effectiveness of opportunistic salpingectomy for ovarian cancer prevention. Gynecol Oncol. 2017;146(2):373-379. doi:10.1016/j.ygyno.2017.05.034 28577884

[22] Dar P, Sachs GS, Strassburger D, Bukovsky I, Arieli S. Ovarian function before and after salpingectomy in artificial reproductive technology patients. Hum Reprod. 2000;15(1):142-144. doi:10.1093/humrep/15.1.142 10611204

[23] Findley AD, Siedhoff MT, Hobbs KA, . Short-term effects of salpingectomy during laparoscopic hysterectomy on ovarian reserve: a pilot randomized controlled trial. Fertil Steril. 2013;100(6):1704-1708. doi:10.1016/j.fertnstert.2013.07.1997 23993887 PMC3844119

[24] Naumann RW, Hughes BN, Brown J, Drury LK, Herzog TJ. The impact of opportunistic salpingectomy on ovarian cancer mortality and healthcare costs: a call for universal insurance coverage. *Am J Obstet Gynecol*. 2021;225(4):397.e1-397.e6. doi:10.1016/j.ajog.2021.03.032 33798477

[25] Reade CJ, McVey RM, Tone AA, . The fallopian tube as the origin of high grade serous ovarian cancer: review of a paradigm shift. J Obstet Gynaecol Can. 2014;36(2):133-140. doi:10.1016/S1701-2163(15)30659-9 24518912

[26] Schenberg T, Mitchell G. Prophylactic bilateral salpingectomy as a prevention strategy in women at high-risk of ovarian cancer: a mini-review. Front Oncol. 2014;4:21. doi:10.3389/fonc.2014.00021 24575389 PMC3918654

[27] Lessard-Anderson CR, Handlogten KS, Molitor RJ, . Effect of tubal sterilization technique on risk of serous epithelial ovarian and primary peritoneal carcinoma. Gynecol Oncol. 2014;135(3):423-427. doi:10.1016/j.ygyno.2014.10.005 25316178 PMC4268072

[28] Madsen C, Baandrup L, Dehlendorff C, Kjaer SK. Tubal ligation and salpingectomy and the risk of epithelial ovarian cancer and borderline ovarian tumors: a nationwide case-control study. Acta Obstet Gynecol Scand. 2015;94(1):86-94. doi:10.1111/aogs.12516 25256594

[29] Falconer H, Yin L, Grönberg H, Altman D. Ovarian cancer risk after salpingectomy: a nationwide population-based study. J Natl Cancer Inst. 2015;107(2):dju410. doi:10.1093/jnci/dju410 25628372

[30] Hanley GE, Pearce CL, Talhouk A, . Outcomes from opportunistic salpingectomy for ovarian cancer prevention. JAMA Netw Open. 2022;5(2):e2147343. doi:10.1001/jamanetworkopen.2021.47343 35138400 PMC8829665

