# Supplementary material for: Salpingectomy and the Risk of Ovarian Cancer in Ontario
Source: JAMA Netw Open. 2023 Aug 11;6(8):e2327198. doi: 10.1001/jamanetworkopen.2023.27198 (PMC10422181; doi:10.1001/jamanetworkopen.2023.27198)
Supplement: Supplement 1. — eMethods. Study Design, Population, and Data; Construction of Cohort; Covariates; Matching; Outcomes; and Statistical Analysis eTable 1. Data Sources and Detailed Variable Definitions eTable 2. Inclusions and Exclusions eTable 3. Descriptive Characteristics of Matched Participants Included in Analysis of Salpingectomy Without Hysterectomy vs No Surgical Procedure eTable 4. Hazard Ratios and 95% CIs of Ovarian Cancer by Analytic Model eTable 5. Hazard Ratios and 95% CIs of Ovarian Cancer by Analytic Model, With Additional Censoring eTable 6. Hazard Ratios and 95% CIs of Ovarian Cancer Among Women With a Bilateral Salpingectomy by Analytic Model eFigure. Cumulative Incidence of Ovarian Cancer in Patients With Tubal Ligation vs No Surgical Procedure [file jamanetwopen-e2327198-s001.pdf]

Supplementary Online Content

Giannakeas V, Murji A, Lipscombe LL, Narod SA, Kotsopoulos J. Salpingectomy and the risk of ovarian cancer in Ontario. *JAMA Netw Open*. 2023;6(8):e2327198. doi:10.1001/jamanetworkopen.2023.27198

eMethods. Study Design, Population, and Data; Construction of Cohort; Covariates; Matching; Outcomes; and Statistical Analysis

eTable 1. Data Sources and Detailed Variable Definitions

eTable 2. Inclusions and Exclusions

eTable 3. Descriptive Characteristics of Matched Participants Included in Analysis of Salpingectomy Without Hysterectomy vs No Surgical Procedure

eTable 4. Hazard Ratios and 95% CIs of Ovarian Cancer by Analytic Model

eTable 5. Hazard Ratios and 95% CIs of Ovarian Cancer by Analytic Model, With Additional Censoring

eTable 6. Hazard Ratios and 95% CIs of Ovarian Cancer Among Women With a Bilateral Salpingectomy by Analytic Model

eFigure. Cumulative Incidence of Ovarian Cancer in Patients With Tubal Ligation vs No Surgical Procedure

This supplemental material has been provided by the authors to give readers additional information about their work.

© 2023 Giannakeas V et al. *JAMA Network Open*.

eMethods. Study Design, Population, and Data; Construction of Cohort; Covariates; Matching; Outcomes; and Statistical Analysis

Study design, population, and data

We conducted a retrospective population-based matched cohort study using healthcare administrative databases in Ontario, Canada. The province of Ontario has a population of 14.5 million residents, all of whom are eligible for healthcare services under the province's universal health coverage. Provincial coverage includes physician consults and procedures, emergency room visits, inpatient hospitalizations, acute care visits, and medication coverage among residents aged 65 and older. Administrative health data in Ontario is housed at ICES, a non-profit organization that provides researchers access to linked health datasets to conduct research studies. ICES is a prescribed entity under §45 of Ontario's Personal Health Information Protection Act, which allows researchers to perform studies without research ethics board approval or the need for informed consent from patients. ICES is an independent, non-profit research institute whose legal status under Ontario's health information privacy law allows it to collect and analyze health care and demographic data, without consent, for health system evaluation and improvement.

ICES data was used to identify women in Ontario with provincial health coverage between January 1st, 1992, and December 31st, 2019. The Registered Persons Database (RPDB) captured demographic information including birthdate, death date, sex, neighbourhood income quintile, and dates of health coverage eligibility. Several datasets were used to capture gynecologic surgeries between 1988 and 2021, these include the Discharge Abstract Database (DAD) for inpatient surgeries, the Same-day Surgery (SDS) database for day surgeries, and the Ontario Health Insurance Plan (OHIP) database for physician billings on surgical procedures. The Ontario Cancer Registry (OCR) was used to identify incident cases of invasive epithelial ovarian, fallopian tube and peritoneal cancer as well as cancer history. Additional datasets used to for descriptive purposes include the National Ambulatory Care Reporting System (NACRS) database for emergency department visits, and the Ontario Drug Benefit (ODB) database for prescription drug dispensations. These datasets were linked using unique encoded patient identifiers and analyzed at ICES. This study was approved by the Research Ethics Board of the Sunnybrook Health Sciences Centre and the Women's College Hospital.

Construction of cohort

Surgical cohorts

Our inclusion cohort consisted of all women residing in Ontario that were between age 18 to 80 between January 1, 1992, and December 31, 2019. Women were observed from the date of cohort entry to their first salpingectomy and/or hysterectomy that was done in an inpatient setting (DAD) or a same-day setting (SDS). The index date was defined as 180 days post-surgery to account for any lead time that would exist because of the surgery, and to avoid occult cancer being an outcome event. Subjects were assigned to one of three mutually exclusive groups based on surgical procedures that occurred in the 180-day post-surgery period: salpingectomy alone, salpingectomy plus hysterectomy, or hysterectomy alone. Subjects were excluded if on the index date: they were ineligible for OHIP coverage at any point in the 2.5 years prior, were under age 18 or over age 80, had a history of cancer, had a history of premalignant conditions or ovarian cysts, had a previous radical gynecologic operation, or had a history of an oophorectomy (Supplemental Table 2).

© 2023 Giannakeas V et al. *JAMA Network Open*.

A tubal ligation cohort was defined using a similar approach to what was described above. Women were observed from the date of cohort entry to their first tubal ligation in the DAD/SDS databases that occurred in the accrual period. The index date was defined as 180 days post-tubal ligation. Women were excluded based on the criteria listed above, or if they had a history of a hysterectomy or salpingectomy surgery.

General population cohort
We sought to identify a cohort of women that could serve as non-surgery control subjects. To do this we randomly assigned index dates to all subjects in the inclusion cohort. Index dates were randomly assigned based on the distribution of index dates among all eligible women in the salpingectomy cohort. Women were excluded from the non-surgery cohort if they had a history of a salpingectomy or hysterectomy prior to their index date. Women in the surgical cohorts were eligible to serve as non-surgical controls if their randomly assigned index date preceded their surgical date.

Covariates
We collected information on a series of variables which describe demographic information, health services utilization, reproductive history, comorbidities, and indications for surgery (among surgical subjects). Demographic variables include age at index date, calendar year, neighbourhood income quintile, residence location (urban, rural), and years eligible for provincial health coverage. Health services utilization variables include history of core primary care visits, specialist visits, inpatient hospitalizations, and emergency department visits. We used the Ontario Mother-Baby linked dataset (MOMBABY) to measure parity as well as any recent delivery hospitalizations. We identified any history of an ovary surgery (non oophorectomy) or sub-total hysterectomy. The Johns Hopkins ACG System software (version 10) was used to capture aggregate diagnosis groups (ADG) based on health services use in the two years prior to a subject's reference date. The reference date is defined as the date of surgery among surgical patients, and 180 days prior to the randomly assigned index date among non-surgical control subjects. The reference date was used to measure health services use prior to any exposure events. Finally, indications for surgery were captured among subjects that had undergone a tubal ligation, salpingectomy, or hysterectomy. The hospitalization record corresponding to the index surgery was used.

Matching
Salpingectomy subjects
Salpingectomy patients were 1:3 matched to women with no gynecologic surgery. Subjects were hard matched on year of index date, age at index date (±2 years), parity, history of tubal ligation, and propensity score. Women that had undergone a salpingectomy alone had different demographic features and surgical indications to women that had undergone a salpingectomy plus hysterectomy. To account for this difference, we ran two separate propensity score models, one for each salpingectomy subgroup (salpingectomy alone, and salpingectomy plus hysterectomy). The propensity score model incorporated income quintile, rurality, years eligible for provincial health coverage, number of primary care visits, history of sub-total hysterectomy, and individual ADGs. Subjects were calliper matched on a value that was 0.2 times the standard deviation of the logit of the propensity score.

© 2023 Giannakeas V et al. *JAMA Network Open*.

We sought to evaluate the association with a surgical comparator group. We performed a second set of matching where salpingectomy patients were 1:1 matched to patients that had undergone a hysterectomy alone. Two separate sets of matches were also performed, one for each salpingectomy subgroup. Propensity score models included variables listed previously as well as variables for surgical indications (abnormal bleeding, endometriosis, fibroids, and pelvic pain).

Tubal ligation subjects

Tubal ligation subjects were 1:3 matched to women with no gynecologic surgery. Tubal ligation subjects were matched using the same methodology and variables described in the salpingectomy cohort, however matched controls could not have a history of tubal ligation.

Outcomes

Primary outcomes

The primary outcome of interest was a diagnosis of incident invasive epithelial ovarian, fallopian tube or peritoneal cancer documented in OCR during the follow-up period. Incident ovarian cancers included any women with ICD 10 codes C56, fallopian tube cancer included ICD 10 codes C57.0 and peritoneal cancer included ICD 10 codes C48.1 and C48.2 (Supplemental Table 1). Matched subjects were followed from their index date to the first of: a primary outcome event, death, end of OHIP eligibility, oophorectomy, or December 31st, 2021.

Tracer outcomes

Tracer events are predefined outcomes that are expected to have no association with the exposure variable. A significant association with these outcomes may indicate the presence of residual confounding or bias. We selected two cancer-related tracer outcomes that we suspect would not be associated with salpingectomy. We used the OCR to capture incident breast cancer (ICD-10 C50) and incident lung cancer (ICD-10 C34) as tracer events. Subjects were followed using the same approach as in the primary outcomes.

Statistical analysis

Baseline descriptive characteristics of the groups were compared using standardized differences. A standardized difference of less than 0.10 was used to determine comparability between the groups for each covariate of interest. Kaplan-Meier analysis was used to estimate the cumulative incidence of cancer among matched subjects. Crude incident rates of cancer were calculated for each group by dividing the number of outcome events by the total number of person-years in the follow-up period. Cox proportional hazards models were used to estimate the adjusted hazard ratio (HR) and 95% confidence intervals (CI) for each exposure group.

Sensitivity analysis

In a sensitivity analysis we censored women that underwent a gynecologic surgery of interest in the follow-up period which may bias the effect estimates. Specifically, no gynecologic surgery controls and tubal ligation subjects were censored if they underwent a salpingectomy or hysterectomy in the follow-up period while women that had undergone a salpingectomy without a hysterectomy were censored if they had a hysterectomy in the follow-up period.

© 2023 Giannakeas V et al. *JAMA Network Open*.

eTable 1. Data Sources and Detailed Variable Definitions

Definition	Data source(s)	Code type	Codes	Conditions
Ovarian cancer (outcome definition)	OCR	ICD-10	C56<br>C57.0<br>C48.1 and C48.2	
Breast cancer (tracer outcome definition)	OCR	ICD-10	C50	
Lung cancer (tracer outcome definition)	OCR	ICD-10	C34	
Hysterectomy (exposure definition) (censor definition)	DAD/SDS	CCP	80.3 – Total abdominal hysterectomy<br>80.4 – Vaginal hysterectomy (subtotal) (total)	
		CCI	1RM89AA – Excision total, uterus and surrounding structures using combined laproscopic and vaginal approach<br>1RM89CA – Excision total, uterus and surrounding structures using vaginal approach<br>1RM89DA – Excision total, uterus and surrounding structures using endoscopic (laproscopic) approach<br>1RM89LA – Excision total, uterus and surrounding structures using open approach	
Salpingectomy (exposure definition) (censor definition)	DAD/SDS	CCP	78.1 – Total salpingectomy (unilateral)<br>78.2 – Total bilateral salpingectomy	
		CCI	1RF89DA – Excision total, fallopian tube using endoscopic (laproscopic) approach<br>1RF89LA – Excision total, fallopian tube using open approach<br>1RF89RA – Excision total, fallopian tube using open vaginal approach	
Subtotal hysterectomy (descriptive event)	DAD/SDS	CCP	80.2 – Subtotal abdominal hysterectomy	
		CCI	1RM87 – Excision partial, uterus and surrounding structures	
		OHIP	S757 – CORPUS UTERI-INC/EXC.-HYSTERECTOMY-TOTAL ABD./VAG.<br>S758 – CORPUS UTERI-INC/EXC.-HYSTERECTOMY-TOTAL-ANT.+POST.REP.	OHIP code must occur in combination with CCP or CCI (± 1 week)

© 2023 Giannakeas V et al. *JAMA Network Open*.

Definition	Data source(s)	Code type	Codes	Conditions
			S759 – CORPUS UTERI-INC/EXC.-HYSTERECTOMY-TOTAL-ANT.ORPOST.REP<br>S763 – CORPUS UTERI INC.EXC.HYSTERECTOMY-RADICAL(WERTHEIMS)<br>S810 – Laparoscopic vaginal hysterectomy<br>S816 – Hysterectomy - vaginal	
Operations on ovary (descriptive event)	DAD/SDS	CCP	77.1 – Local excision or destruction of lesion or tissue of ovary<br>77.6 – Repair of ovary<br>77.7 – Freeing of adhesions of ovary and fallopian tube<br>77.8 – Invasive diagnostic procedures on ovary<br>77.9 – Other operations on ovary	
		CCI	1RB52 – Drainage, ovary NEC<br>1RB55 – Removal of device, ovary NEC<br>1RB56 – Removal of foreign body, ovary NEC<br>1RB57 – Extraction, ovary NEC<br>1RB58 – Procurement, ovary NEC<br>1RB59 – Descruction, ovary NEC<br>1RB74 – Fixation, ovary NEC<br>1RB80 – Repair, ovary NEC<br>1RB83 – Transfer, ovary NEC<br>1RB85 – Transplant, ovary NEC<br>1RB87 – Excision partial, ovary NEC<br>1RD52 – Drainage, ovary with fallopian tube<br>1RD72 – Release, ovary with fallopian tube	
Operations on fallopian tube (descriptive event)		CCP	78.3 – Bilateral endoscopic destruction or occlusion of fallopian tubes<br>78.4 – Other bilateral destruction or occlusion of fallopian tubes<br>78.5 – Other salpingectomy<br>78.6 – Repair of fallopian tube<br>78.7 – Insufflation of fallopian tube<br>78.8 – Invasive diagnostic procedures on fallopian tube<br>78.9 – Other operations on fallopian tubes	
		CCI	1RF50 – Dilation, fallopian tube<br>1RF51 – Occlusion, fallopian tube<br>1RF52 – Drainage, fallopian tube NEC<br>1RF55 – Removal of device, fallopian tube NEC<br>1RF56 – Removal of foreign body, fallopian tube NEC<br>1RF59 – Destruction, fallopian tube NEC<br>1RF72 – Release, fallopian tube NEC<br>1RF74 – Fixation, fallopian tube NEC	

© 2023 Giannakeas V et al. *JAMA Network Open*.

Definition	Data source(s)	Code type	Codes	Conditions
			1RF80 – Repair, fallopian tube NEC<br>1RF83 – Transfer, fallopian tube NEC<br>1RF87 – Excision partial, fallopian tube NEC	
Tubal ligation (exclusion and censoring event)	DAD/SDS	CCP	78.41 – Other bilateral ligation and crushing of fallopian tubes<br>78.42 – Other bilateral ligation and division of fallopian tubes<br>78.49 – Other bilateral destruction or occlusion of fallopian tubes nec	
		CCI	1RF51 – Occlusion, fallopian tube endoscopic [laparoscopic]	
		OHIP	S741 – FALLOPIAN TUBE-OCCL/INTERRUP/REM.ANY METHOD STERILIZ.	
Radical hysterectomy (exclusion)	DAD/SDS	CCP	80.5 – Radical abdominal hysterectomy<br>80.6 – Radical vaginal hysterectomy<br>80.7 – Pelvic evisceration	
		CCI	1RM91AA – Excision, radical, uterus and surrounding structures. Using combined laparoscopic and vaginal approach (includes laparoscopic radical vaginal hysterectomy)<br>1RM91CA – Excision, radical, uterus and surrounding structures. Using vaginal approach<br>1RM91DA – Excision, radical, uterus and surrounding structures. Using endoscopic (laparoscopic) approach<br>1RM91LA – Excision, radical, uterus and surrounding structures. Using abdominal approach (e.g. Wertheim operation) (includes modified radical hysterectomy)	
Oophorectomy (exclusion) (censoring event)	DAD/SDS	CCP	77.0 – Oophorotomy<br>77.2 – Unilateral oophorectomy<br>77.3 – Unilateral salpingo-oophorectomy<br>77.4 – Bilateral oophorectomy<br>77.5 – Bilateral salpingo-oophorectomy	
		CCI	1RB89 – Excision total, ovary NEC<br>1RD89 – Excision total, ovary with fallopian tube	
	OHIP	Feecode	S738 – FALLOP.TUBE-EXC.SUT.REP-SALPINGECTOMY&SALP/OOPHOREC.UNIL/BIL<br>S745 – OVARY.EXC.OOPHORECTOMY/OOPHOROCYSTECTOMY<br>S782 – OVARY-EXC.-OOPHORECTOMY WITH TOTAL OMENTECTOMY	
Endometriosis	DAD/SDS	ICD-9	617*	
		ICD-10	N80*	
Uterine leiomyoma	DAD/SDS	ICD-9<br>ICD-10	218*<br>D25*	
	DAD/SDS	ICD-9	219.9*	

© 2023 Giannakeas V et al. *JAMA Network Open*.

Definition	Data source(s)	Code type	Codes	Conditions
Benign uterine neoplasm		ICD-10	D26*	
Benign ovarian cyst/masses	DAD/SDS	ICD-9	220*, 6200*, 6201*, 6202*, 6205*	
		ICD-10	D27*, N830*, N831*, N832*, N835*	
Abnormal bleeding	DAD/SDS	ICD-9	626*	
		ICD-10	N92*, N93*	
Pelvic inflammatory disease	DAD/SDS	ICD-9	614.3 – 614.9*, 615*, 616*	
		ICD-10	N71*, N72*, N73*, N74*, N75*, N76*, N77*	
Hydrosalpinx	DAD/SDS	ICD-9	614.0, 614.1, 614.2	
		ICD-10	N70.0, N70.1	
Prolapse	DAD/SDS	ICD-9	618*	
		ICD-10	N81*	
Ectopic pregnancy	DAD/SDS	ICD-9	633*	
		ICD-10	O00*	

The following administrative databases using unique encoded patient identifiers were included in the analysis: i) Registered Persons Database (RPDB) for patient demographics; ii) Canadian Institute for Health Information (CIHI) Discharge Abstract Database (DAD) for inpatient surgeries; iii) CIHI Same-day Surgery (SDS) database for outpatient procedures; iv) Ontario Health Insurance Plan (OHIP) database for physician billings; v) Ontario Cancer Registry (OCR) to identify patients with a cancer diagnosis; vi) CIHI National Ambulatory Care Reporting System (NACRS) database for emergency department visits; vii) Ontario Drug Benefit (ODB) database for prescription drug dispensations in patients >65 years of age.
Other abbreviations: CCI (Canadian Classification of Health Interventions); CCP (Canadian Classification of Diagnostic, Therapeutic, and Surgical Procedures); ICD (International Classification of Diseases)

© 2023 Giannakeas V et al. *JAMA Network Open*.

eTable 2. Inclusions and Exclusions

	**Unexposed**		**Exposed**		
**Exclusions n (%)**	**No surgery**	**Hysterectomy**	**Tubal ligation**	**Salpingectomy *with* hysterectomy**	**Salpingectomy *without* hysterectomy**
1. Death date prior to/on index date	718,137	2,291	117	41	81
	8.5	0.5	0	0.1	0.3
2. OHIP ineligible on index date, or < 2.5 years from index date	2,148,757	14,687	11,451	733	1,079
	25.3	3.3	4.5	2.1	4.2
3. Age <18 on index date	726,516	46	13	7	168
	8.6	0	0	0	0.7
4. Age >80 on index date	300,452	9,218	*SS*	115	116
	3.5	2.1	0	0.3	0.5
5. History of gynecologic cancer (in the OCR) prior to/on index date	29,709	53,898	1,917	2,402	990
	0.4	12.3	0.8	6.7	3.9
6. History of other cancer (in the OCR) prior to/on index date	153,811	17,517	1,882	874	590
	1.8	4	0.7	2.4	2.3
7. History of premalignant conditions prior to/ on index date	132,488	45,752	13,431	3,297	1,193
	1.6	10.4	5.3	9.2	4.7
8. History of ovarian cyst(s) prior to/ on index date	102,587	55,864	11,704	4,613	5,017
	1.2	12.7	4.6	12.9	19.7
9. History of hysterectomy prior to reference date	33,309	n/a	*SS*	n/a	66
	0.4		0		0.3
10. History of salpingectomy prior to reference date	980	187	29	n/a	n/a

© 2023 Giannakeas V et al. *JAMA Network Open*.

	Unexposed		Exposed		
**Exclusions n (%)**	**No surgery**	**Hysterectomy**	**Tubal ligation**	**Salpingectomy *with* hysterectomy**	**Salpingectomy *without* hysterectomy**
	0	0	0		
11. History of radical gynecologic operation prior to/on index date	471	15	*SS*	*SS*	71
	0	0	0	0	0.3
12. History of oophorectomy operation prior to/on index date	24,267	80,784	2,181	2,830	2,612
	0.3	18.4	0.9	7.9	10.3
Eligible for study	*4,111,882*	*158,787*	*209,026*	*20,842*	*13,451*
	48.5	*36.2*	*83*	*58.3*	*52.9*

SS: data suppression due to small cell counts (<6)
OHIP (Ontario Health Insurance Plan); OCR (Ontario Cancer Registry)

© 2023 Giannakeas V et al. *JAMA Network Open*.

eTable 3. Descriptive Characteristics of Matched Participants Included in Analysis of Salpingectomy Without Hysterectomy vs No Surgical Procedure

Variable	Value	Total	No surgery	Salpingectomy without a hysterectomy	Std diff
Overall		51,144 (100%)	38,358 (75.0%)	12,786 (25.0%)	
Calendar year (cont.)	Mean (SD)	2011.9 (8.3)	2011.9 (8.3)	2011.9 (8.3)	0
	Median (IQR) [Range]	2016.0 (2007.0-2018.0) [1992.0-2020.0]	2016.0 (2007.0-2018.0) [1992.0-2020.0]	2016.0 (2007.0-2018.0) [1992.0-2020.0]	
Age (cont.)	Mean (SD)	38.8 (7.9)	38.8 (7.9)	38.7 (8.0)	0
	Median (IQR) [Range]	38.1 (33.5-43.2) [18.0-79.9]	38.1 (33.6-43.2) [18.0-79.9]	38.1 (33.5-43.2) [18.0-79.8]	
Neighborhood income quintile	1 - Low	11,164 (21.8%)	8,426 (22.0%)	2,738 (21.4%)	0.01
	2	10,482 (20.5%)	7,829 (20.4%)	2,653 (20.7%)	0.01
	3	10,298 (20.1%)	7,738 (20.2%)	2,560 (20.0%)	0
	4	10,417 (20.4%)	7,808 (20.4%)	2,609 (20.4%)	0
	5 - High	8,783 (17.2%)	6,557 (17.1%)	2,226 (17.4%)	0.01
Residence location	Urban	44,526 (87.1%)	33,394 (87.1%)	11,132 (87.1%)	0
	Rural	6,618 (12.9%)	4,964 (12.9%)	1,654 (12.9%)	0
Time eligible in OHIP (years)	Mean (SD)	18.1 (9.1)	18.1 (9.0)	18.2 (9.2)	0.01
	Median (IQR) [Range]	19.7 (9.5-26.7) [2.5-30.2]	19.5 (9.6-26.7) [2.5-30.2]	20.4 (9.2-26.9) [2.5-30.2]	
Core primary care visits to GP/FP (cont.)	Mean (SD)	3.9 (4.1)	3.9 (4.2)	3.8 (4.0)	0.02
	Median (IQR) [Range]	3.0 (1.0-5.0) [0.0-112.0]	3.0 (1.0-5.0) [0.0-106.0]	3.0 (1.0-5.0) [0.0-112.0]	
Urgent inpatient hospitalization episodes (cat.)	0	46,717 (91.3%)	35,232 (91.9%)	11,485 (89.8%)	0.07
	1	3,577 (7.0%)	2,558 (6.7%)	1,019 (8.0%)	0.05
	2	601 (1.2%)	398 (1.0%)	203 (1.6%)	0.05
	3+	249 (0.5%)	170 (0.4%)	79 (0.6%)	0.02
Delivery hospitalizations captured in MOMBABY (ever) (cont.)	Mean (SD)	1.4 (1.3)	1.4 (1.3)	1.4 (1.3)	0

© 2023 Giannakeas V et al. *JAMA Network Open*.

Variable	Value	Total	No surgery	Salpingectomy without a hysterectomy	Std diff
	Median (IQR) [Range]	1.0 (0.0-2.0) [0.0-8.0]	1.0 (0.0-2.0) [0.0-8.0]	1.0 (0.0-2.0) [0.0-8.0]	
Any tubal ligation prior to index date	Yes	3,224 (6.3%)	2,418 (6.3%)	806 (6.3%)	0
Any subtotal hysterectomy prior to index date	Yes	11,950 (23.4%)	8,820 (23.0%)	3,130 (24.5%)	0.03
Any ovary surgery prior to index date	Yes	805 (1.6%)	431 (1.1%)	374 (2.9%)	0.13
Aggregate diagnosis groups (cont.)	Mean (SD)	6.9 (3.0)	6.9 (3.1)	6.9 (3.0)	0.01
	Median (IQR) [Range]	7.0 (5.0-9.0) [0.0-23.0]	7.0 (5.0-9.0) [0.0-23.0]	7.0 (5.0-9.0) [0.0-21.0]	
Aggregate diagnosis groups (cat.)	0 – 4	11,749 (23.0%)	8,917 (23.2%)	2,832 (22.1%)	0.03
	5 – 9	29,689 (58.0%)	22,108 (57.6%)	7,581 (59.3%)	0.03
	10+	9,706 (19.0%)	7,333 (19.1%)	2,373 (18.6%)	0.01
Follow-up time (years)	Mean (SD)	8.6 (7.7)	8.7 (7.7)	8.4 (7.5)	0.05
	Median (IQR) [Range]	5.6 (3.1-11.0) [0.0-29.2]	5.6 (3.1-11.3) [0.0-29.2]	5.4 (3.0-10.3) [0.1-29.2]	
Any ovarian, fallopian or peritoneal cancer diagnosis in the follow-up period	Yes	64 (0.1%)	48 (0.1%)	16 (0.1%)	0
Any breast cancer diagnosis in the follow-up period	Yes	701 (1.4%)	536 (1.4%)	165 (1.3%)	0.01
Any lung cancer diagnosis in the follow-up period	Yes	151 (0.3%)	119 (0.3%)	32 (0.3%)	0.01

Abbreviations: IQR (interquartile range); Std diff (standardized difference); OHIP (Ontario Health Insurance Plan); GP (general practitioner); FP (family practitioner)

Similar tables are available for other matched cohorts.

© 2023 Giannakeas V et al. *JAMA Network Open*.

eTable 4. Hazard Ratios and 95% CIs of Ovarian Cancer Only by Analytic Model

Model	n	Person-years	Events	Rate (per 100,000 PY)	HR (95% CI)	*P*-value
Model 1						
No surgery	98,637	742,872	110	14.81	1.00 (Reference)	
All salpingectomy	32,879	242,692	29	11.95	0.81 (0.54-1.22)	0.3167
Model 2						
Hysterectomy alone	21,724	199,986	31	15.5	1.00 (Reference)	
All salpingectomy	21,724	196,345	26	13.24	0.86 (0.51-1.45)	0.5786
Model 3						
No surgery	425,094	5,341,614	528	9.88	1.00 (Reference)	
Tubal ligation	141,698	1,767,152	134	7.58	0.77 (0.64-0.93)	0.0067

Ovarian cancer includes invasive epithelial ovarian cancer *only* (excludes fallopian tube cancer and primary peritoneal cancer)
Abbreviations: PY (person-years); CI (confidence interval); HR (hazard ratio)

© 2023 Giannakeas V et al. *JAMA Network Open*.

eTable 5. Hazard Ratios and 95% CIs of Ovarian Cancer by Analytic Model, With Additional Censoring

**Model**	**n**	**Person-years**	**Events**	**Rate (per 100,000 PY)**	**HR (95% CI)**	***P*-value**
Model 1						
No surgery	98,637	720,292	113	15.69	1.00 (Reference)	
All salpingectomy	32,879	237,568	31	13.05	0.84 (0.56-1.24)	0.3779
Model 2						
Hysterectomy alone	21,724	199,787	33	16.52	1.00 (Reference)	
All salpingectomy	21,724	191,969	28	14.59	0.90 (0.54-1.49)	0.6824
Model 3						
No surgery	425,094	4,909,999	514	10.47	1.00 (Reference)	
Tubal ligation	141,698	1,669,897	135	8.08	0.77 (0.64-0.93)	0.0074

Ovarian cancer includes invasive epithelial ovarian cancer, fallopian tube cancer and primary peritoneal cancer.
Abbreviations: PY (person-years); CI (confidence interval); HR (hazard ratio)
Additional censoring for gynecologic surgery (see Methods for detail).

© 2023 Giannakeas V et al. *JAMA Network Open*.

eTable 6. Hazard Ratios and 95% CIs of Ovarian Cancer Among Women With a Bilateral Salpingectomy by Analytic Model

Model	n	PY	Follow-up, mean (range), y	Events	Rate (per 100,000 PY)	HR (95% CI)	*P*-value
Model 1							
No surgery	76,227	397,026	5.2 (0-20.0)	60	15.11	1.00 (Reference)	
Bilateral salpingectomy	25,409	131,657	5.2 (0-19.2)	11	8.36	0.55 (0.29-1.05)	0.072
Model 2							
Hysterectomy alone	15,526	96,436	6.2 (0-20.0)	12	12.44	1.00 (Reference)	
Bilateral salpingectomy	15,526	96,080	6.2 (0-19.2)	9	9.37	0.75 (0.32-1.79)	0.5236

Ovarian cancer includes invasive epithelial ovarian cancer, fallopian tube cancer and primary peritoneal cancer.
Abbreviations: PY (person-years); CI (confidence interval); HR (hazard ratio)

© 2023 Giannakeas V et al. *JAMA Network Open*.

eFigure. Cumulative Incidence of Ovarian Cancer in Patients With Tubal Ligation vs No Surgical Procedure

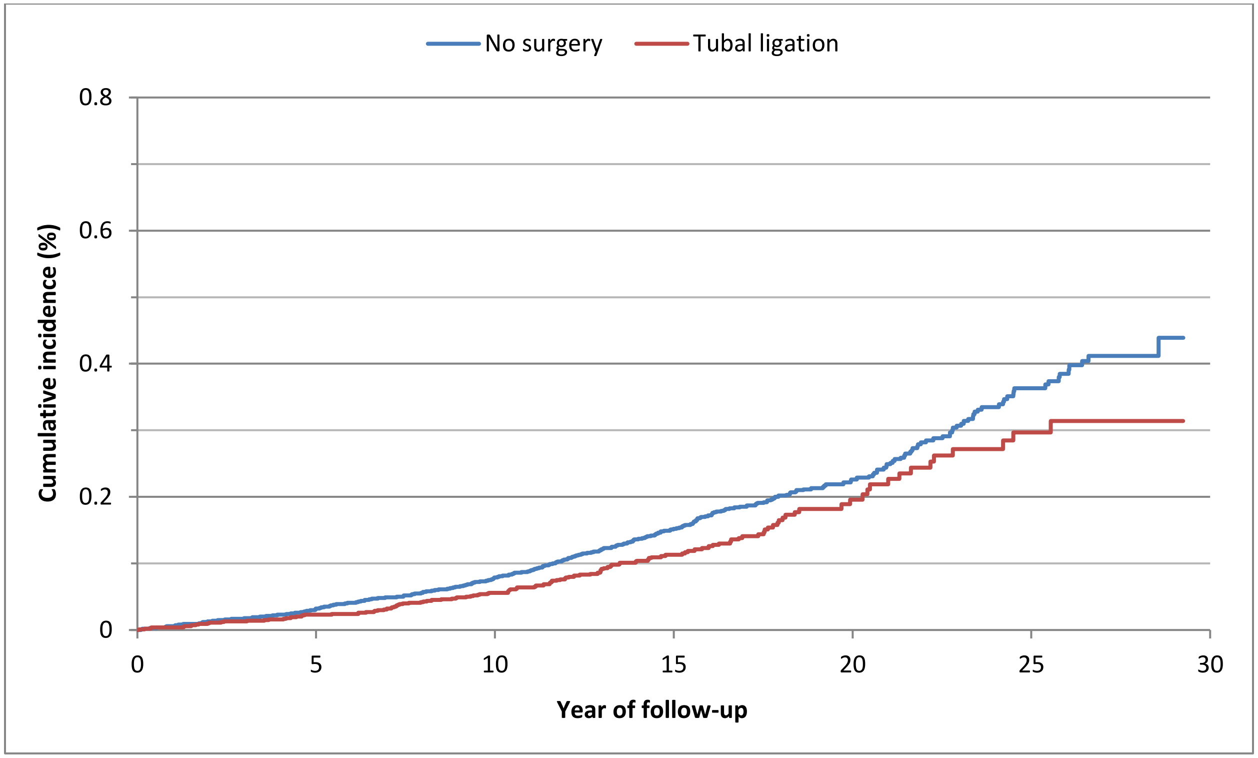

© 2023 Giannakeas V et al. *JAMA Network Open*.
