# Supplementary material for: Salpingectomy and the Risk of Ovarian Cancer in Ontario
Source: JAMA Netw Open. 2023 Aug 11;6(8):e2327198. doi: 10.1001/jamanetworkopen.2023.27198 (PMC10422181; doi:10.1001/jamanetworkopen.2023.27198)
Supplement: Supplement 2. — Data Sharing Statement [file jamanetwopen-e2327198-s002.pdf]

Data Sharing Statement

Giannakeas. Salpingectomy and the Risk of Ovarian Cancer in Ontario. *JAMA Netw Open*. Published August 11, 2023. doi:10.1001/jamanetworkopen.2023.27198

Data
Data available: No

Additional Information
Explanation for why data not available: This study makes use of provincial administrative data.
